# Neuroticism, Physical Activity, and Cognitive Functioning in a Population-Based Cohort of Older Adults

**DOI:** 10.21203/rs.3.rs-2874085/v1

**Published:** 2023-05-18

**Authors:** Pankaja Desai, Todd Beck, Kristin Krueger, Robert Wilson, Denis Evans, Kumar Rajan

**Affiliations:** Rush University Medical Center; Rush University Medical Center; Rush University Medical Center; Rush University Medical Center; Rush University Medical Center; Rush University Medical Center

**Keywords:** Neuroticism, Physical Activity, Cognitive Function, Cognitive Decline

## Abstract

**Background.:**

Little is known about how physical activity influences the relationship between neuroticism and cognitive function and cognitive decline.

**Methods.:**

Data from the Chicago Health and Aging Project (CHAP) was utilized to conduct this study. CHAP is a population-based cohort study of chronic conditions in older adults. Participants completed in-home interviews cycles of three years from 1993–2012. Mixed effects regression models were conducted to test the associations between physical activity, neuroticism, and the interaction between neuroticism and global cognitive function and global cognitive decline. Stratified mixed effects regression models by physical activity level were conducted to test the associations between neuroticism and global cognitive function and global cognitive decline.

**Results.:**

A total of 7,685 participants were eligible for this study. Participants were 62% female and 64% African American. We found statistically significant associations for the interaction of medium physical and neuroticism (β = 0.014 (SE = 0.007), p = .037) and the interaction of high physical activity and neuroticism (β = 0.021 (SE = 0.007), p = .003) on global cognitive function at baseline but not for decline over time. Stratified analysis showed that among participants with high physical activity levels, the association between neuroticism and global cognitive decline was statistically significant (β=−0.002 (SE = 0.001), p = .023).

**Conclusion.:**

Increasing physical activity level benefits the cognitive functioning of individuals with high neuroticism. Interventions should incorporate health behavior change approaches which aim to reduce characteristics of neuroticism.

## Introduction

Neuroticism is an established personality trait that is an important target for public health interventions.^1^ It is strongly correlated with and predicts several physical and mental health conditions and comorbidities, as well as health and mental health care utilization.^2^ Neuroticism is defined by characteristics such as anxiety, depression, anger, variability of emotion, susceptibility to irritation, and greater self-consciousness. Individuals with more neuroticism tend to have difficulty managing stress and often experience feelings of being threatened, overwhelmed, and hopeless during day-to-day life occurrences. When individuals experience symptoms of depression and anxiety that are clinically noteworthy, this may signify the interaction of neuroticism with a stressor.^1^

Less neuroticism is associated with increased physical activity participation and decreased sedentary behavior and inactivity.^3^ Individuals with an inactive lifestyle are more likely to develop negative personality traits over time.^4^ Greater neuroticism is also associated with cognitive decline and poor cognitive performance.^5^ Limited information exists regarding the relationship between neuroticism, physical activity and cognitive decline. Therefore, in this study, we will test the interaction of physical activity and neuroticism on cognitive function and decline over time.

## Methods

We analyzed data from the Chicago Health and Aging Project (CHAP), which is a population-based cohort study that examines health conditions in community-dwelling African American and White older adults. Recruitment of study participants occurred using door-to-door census. In-home interviews were completed in three-year cycles from 1993 to 2012. CHAP has a total of 10,802 participants. Clinical evaluations were completed with a stratified, random sample of approximately 33% of total CHAP participants. CHAP study design is further described elsewhere.^6^ A total of 7,685 participants were eligible for this study and completed at least two time points of cognitive performance measurement. CHAP is approved by the Rush University Medical Center IRB, and CHAP participants provided written informed consent.

### Neuroticism

Neuroticism was measured using items from the NEO Five-Factor Inventory. Participants were asked to rate each of the following items on a scale of 1 (*Strongly disagree*) to 5 (*Strongly agree*): *I often feel tense and jittery; I am not a worrier; I often feel helpless and want someone else to solve my problems; I often get angry at the way people treat me*. Ratings were summed to calculate a total score between 4–20 and recoded 0–16. The second item was reverse coded.^7^ For the figure only, neuroticism was categorized as low at approximately the 10th percentile and high at approximately the 90th percentile.

### Physical Activity

Physical activity participation was measured using items from the 1985 US Health Interview Survey. Participants reported their participation in the following activities *in the past 14 days*: *walking for exercise, jogging or running, gardening or yard work, dancing, calisthenics or general exercise, golf, bowling, bicycle riding, swimming or water exercises*, and *other exercises, sports or physically active hobbies*.^8–9^ Frequency was multiplied by the number of minutes for each activity. This total was divided by two to determine the number of minutes per week or seven days per activity. The minutes per activity were then summed across all items to obtain the total number of minutes per week of physical activity participation. Physical activity was categorized into little activity, medium activity, and high activity. Participants with little activity responded to at least four items and had 0 minutes per week of participation. Participants with medium activity engaged in a total of less than or equal to150 minutes per week, and participants with high activity engaged in a total which was greater than 150 minutes per week. Physical activity was categorized because of the large number of participants with little activity.

### Demographic Characteristics and Chronic Medical Conditions

Demographic characteristics include age, sex, race, and education level and were obtained using items from the 1990 US Census Bureau.^10^ Chronic medical conditions consist of diabetes, stroke, hypertension, cancer, fractured or broken hip, and cardiovascular conditions.^11–12^

### Cognitive Function

Cognitive functioning was assessed utilizing the East Boston Memory Test: Immediate Recall and Delayed Recall, which measures episodic memory, the Symbol Digit Modalities Test (modified, oral version), which measures perceptual speed, and the Mini-Mental State Examination (MMSE).^13–16^ The z-score for each measure was determined using the mean and standard deviation of the baseline score. Global cognitive function score was calculated by taking the mean of z-scores from all measures.^17^

### Statistical Analysis

Descriptive analysis was conducted in total and by physical activity category. Boxplots were developed to show the median level of neuroticism by physical activity category at baseline. Mixed effects regression models were conducted to test the associations between neuroticism as a continuous variable and physical activity as a categorical variable on the longitudinal trajectory of global cognitive function. Initially, two models were fit to examine the effects neuroticism and physical activity levels separately. A third model then included both effects into one model and finally a fourth model added a neuroticism by physical activity interaction term. Mixed effects regression models were also conducted stratified by the three levels of physical activity. Models adjusted for age, race, sex, education, medical conditions, time, and interactions of each with time. Level of statistical significance used for this analysis was p < .05. Analysis was done using SAS. A figure was developed in R to plot global cognitive decline over time among participants with high neuroticism, little physical activity, high neuroticism, high physical activity, low neuroticism, little physical activity, and low neuroticism, high physical activity.

## Results

### Descriptive Analysis.

Table 1 describes baseline characteristics of the study sample in total and by physical activity category. The total study sample had a mean age of 72 years, included 62% female participants and 64% African American participants, and had a mean of 12 years of education. At baseline, the mean neuroticism score was 5.35 out of 16, and the mean minutes of physical activity per week was 75. Participants with little physical activity had a mean neuroticism score of 5.77 compared to a mean of 5.01 for participants with high physical activity.

### Mixed Effects Regression Models.

Table 2 includes the results of four models of longitudinal global cognitive function examining associations between 1. neuroticism, 2. physical activity, 3. neuroticism and physical activity, and 4. the interaction of neuroticism and physical. Betas that do not include time represent units of cognitive function at baseline. Terms that include time are interpreted as units of annual rate of change in cognitive function. Model 1 shows statistically significant associations between neuroticism and baseline global cognitive function (β=−0.041 (SE = 0.003), p = .000) and global cognitive decline (β=−0.002 (SE = 0.001), p = .002). Model 2 has statistically significant associations between medium physical activity and baseline level (β = 0.041 (SE = .016), p = .001) and decline (β = 0.005 (SE = 0.003), p = .074) and between high physical activity and baseline level (β = 0.051(SE = .016), p = .002) and decline (β = 0.008(SE = 0.003), p = .003). Model 3 indicates statistically significant associations between neuroticism and baseline level (β= −0.040 (SE = 0.003), p = .000) and decline (β= −0.001 (SE = 0.001), p = .004), between high physical activity and baseline level (β = 0.033 (SE = 0.016), p = .041) and decline (β = 0.008 (SE = 0.006), p = .003), but not for medium physical activity for either baseline level or decline. Model 4 shows that the interaction of medium physical and neuroticism (β = 0.014 (SE = 0.007), p = .037) and the interaction of high physical activity and neuroticism (β = 0.021 (SE = 0.007), p = .003) on baseline global cognitive function were statistically significant. We did not find statistically significant associations between the interaction of medium physical activity and neuroticism or high physical activity and neuroticism on global cognitive decline.

Table 3 shows associations between neuroticism and global cognitive function, stratified by physical activity level. There are inverse associations between neuroticism and baseline cognitive function that are statistically significant within each physical activity level: little (β=−0.048 (SE = .006), p = .000), medium (β=−0.038 (SE = .005), p = .000), and high (β=−0.036 (SE = .005), p = .000). Among participants with high physical activity, we found a statistically significant association between neuroticism and global cognitive decline (β=−0.002 (SE = 0.001), p = .023). For individuals with medium activity, we did not find statistically significant associations between neuroticism and global cognitive decline, (β=−0.002 (SE = 0.001), p = 0.053).

**Figure 1:**
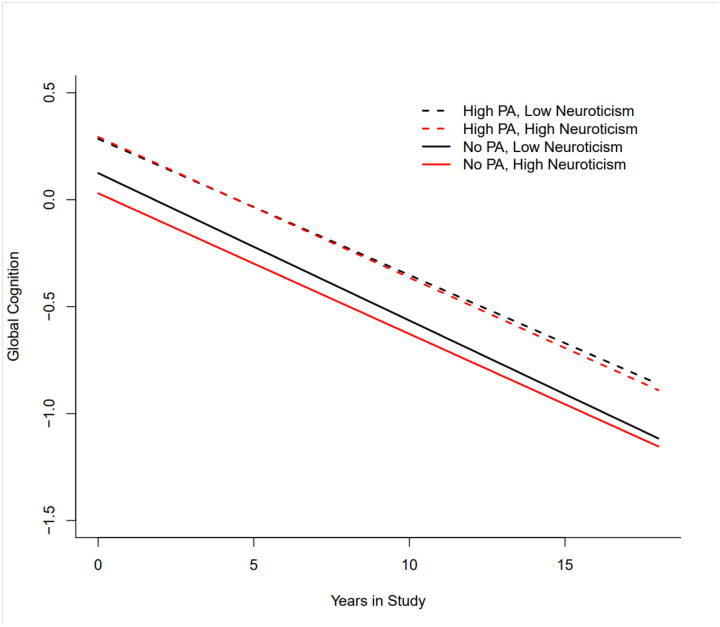
Global Cognitive Decline by Physical Activity and Neuroticism Level

Figure 1 describes global cognitive decline over the number of years in the study. The figure shows that individuals who have high or low levels of neuroticism who engage in high physical activity level begin at a similar level of cognitive function which is higher than for individuals who engage in little physical activity. Participants with high or low levels of neuroticism have a rate of global cognitive decline that is similar and is a slower rate compared to individuals who engage in little physical activity.

## Discussion

Study findings indicate that the association between neuroticism and global cognitive function is dependent on physical activity level. A high level of physical activity attenuates the association between neuroticism and global cognitive decline.

Little work has been done to examine how physical activity, which is a modifiable risk factor, influences the relationship between neuroticism and cognitive decline in older adults. However, studies have been conducted which evaluate the role of physical activity in the associations between characteristics of neuroticism, such as depression or anxiety, and cognitive decline. Data from the National Health and Nutrition Examination Survey (2011–2014) was used to assess how physical activity impacts the relationship between depression and cognitive function in older adults. Results showed that participating in 150 minutes per week of physical activity at moderate to vigorous intensity helps to modify the association between depression and cognitive function and that physical activity may protect cognitive function from depression.^18^ The English Longitudinal Study of Ageing (ELSA) found that decreased physical activity served as a mediator in the relationship between depression and cognitive decline.^19^

More research has focused on evaluating associations between depressive symptoms and Alzheimer’s disease compared to symptoms of anxiety. Yet, anxiety is approximately 40% prevalent in individuals with Alzheimer’s disease. Symptoms of anxiety can also occur in early stages of experiencing cognitive impairment and can exacerbate symptoms, especially if management strategies are not in place.^20^ Data from the Mayo Clinic Study of Aging was utilized to evaluate the longitudinal relationships between physical inactivity and neuropsychiatric symptoms and incidence of mild cognitive impairment. Findings indicated an interaction that was additive of physical inactivity, disruption of sleep, clinically diagnosed depression or clinically diagnosed anxiety on MCI incidence.^21^ Another study focused on participants with recent Parkinson’s Disease diagnosis who were part of the Parkinson’s Progression Marker Initiative (PPMI). Study results showed that having anxiety in the early part of the disease trajectory may lead to decreased engagement in physical activity and in turn, contribute to cognitive decline.^22^

However, the relationship between neuroticism and physical activity is complex. Healthy neuroticism is described as neuroticism interacting with conscientiousness. In specific situations, neuroticism can benefit health. Analysis of data from the studies in the Integrative Analysis of Longitudinal Studies of Aging and Dementia (IALSA) network found healthy neuroticism to be predictive of increased physical activity participation and that conscientiousness may weaken the relationship between neuroticism and physical activity.^23^

There are several limitations to this study. Physical activity was self-reported, and intensity of physical activity was not measured. We also did not measure healthy neuroticism. There are only two races represented in the study. Finally, a subset of total CHAP participants were selected for this study.

In future work, we will examine associations by type of physical activity as well focus on specific items in the neuroticism measure. We also plan to test for race, sex, and education differences in the associations over time. Additional research is needed to understand the frequency, intensity, and types of physical activity, as well health behavior change strategies needed, to reduce the impact of neuroticism on cognitive decline.

## Figures and Tables

**Table 1:** Baseline Sample Characteristics in Total and by Physical Activity Category

Variable	Overall N = 7,685^1^	0 Minutes N = 2285^1^	<=150 Minutes N = 2585^1^	>150 Minutes N = 2815^1^
Age	72.2 (6.2)	72.6 (6.7)	72.4 (6.3)	71.7 (5.6)
Education	12.4 (3.5)	11.6 (3.4)	12.4 (3.5)	13.1 (3.6)
Medical Conditions	1.11 (0.98)	1.23 (1.03)	1.11 (0.98)	1.01 (0.93)
Global cognition	0.30 (0.70)	0.15 (0.78)	0.30 (0.67)	0.41 (0.62)
Episodic Memory	0.29 (0.83)	0.17 (0.91)	0.29 (0.80)	0.39 (0.76)
Perceptual Speed	0.35 (0.93)	0.17 (0.93)	0.36 (0.92)	0.50 (0.91)
Neuroticism	5.35 (2.27)	5.77 (2.38)	5.34 (2.26)	5.01 (2.12)
Physical Activity (min)	75 (0, 240)	0 (0, 0)	60 (30, 105)	345 (225, 540)
Study Time (yrs)	6.9 (3.5, 9.6)	6.5 (3.4, 9.3)	7.1 (3.5, 9.8)	7.0 (3.6, 10.1)
Female	4,803 (62%)	1,638 (72%)	1,670 (65%)	1,495 (53%)
Black	4,929 (64%)	1,723 (75%)	1,670 (65%)	1,536 (55%)

1 Mean (SD); Median (IQR); n (%)

**Table 2:** Associations Between Physical Activity, Neuroticism, and Global Cognitive Function and Decline

	Model 1			Model 2			Model 3			Model 4		
Participants	7691			7685			7685			7685		
Total Observations	25689			25676			25676			25676		
	β	SE	p-value	β	SE	p-value	β	SE	p-value	β	SE	p-value
neuroticism	−0.041	0.003	0.000				−0.040	0.003	0.000	−0.052	0.005	0.000
neuroticism*time	−0.002	0.001	0.002				−0.001	0.001	0.004	−0.001	0.001	0.396
medium_pa				0.041	0.016	0.011	0.031	0.016	0.057	0.021	0.017	0.207
high_pa				0.051	0.016	0.002	0.033	0.016	0.041	0.024	0.017	0.143
medium_pa*time				0.005	0.003	0.074	0.005	0.003	0.098	0.005	0.003	0.071
high_pa*time				0.008	0.003	0.003	0.008	0.003	0.006	0.008	0.003	0.004
medium_pa*neuroticism										0.014	0.007	0.037
high_pa*neuroticism										0.021	0.007	0.003
medium_pa*neuroticism*time										−0.001	0.001	0.443
high_pa*neuroticism*time										−0.001	0.001	0.383

Models adjusted for age, sex, race, education, medical conditions, and each of their interactions with time

**Table 3:** Stratified Analysis by Physical Activity Level: Associations Between Neuroticism and Global Cognitive Function and Decline

	Little Activity			Medium Activity			High Activity		
Total Observations	2285			2585			2815		
Particivants	7232			8753			9691		
Neuroticism	−0.048	0.006	0.000	−0.038	0.005	0.000	−0.036	0.005	0.000
Neuroticism*time	−0.001	0.001	0.358	−0.002	0.001	0.053	−0.002	0.001	0.023

Models adjusted for age, sex, race, education, medical conditions, and each of their interactions with time

## Data Availability

The datasets used and/or analysed during the current study available from the corresponding author on reasonable request. Please contact the corresponding author to make a request.
